# Anti-GARP Antibodies Inhibit Release of TGF-β by Regulatory T Cells via Different Modes of Action, but Do Not Influence Their Function In Vitro

**DOI:** 10.4049/immunohorizons.2200072

**Published:** 2023-03-16

**Authors:** Frederik H. Igney, Rebecca Ebenhoch, Felix Schiele, Herbert Nar

**Affiliations:** * Discovery Research, Cancer Immunology & Immune Modulation, Boehringer Ingelheim Pharma GmbH & Co. KG, Biberach an der Riss, Germany; † Discovery Research, Structural Research, Boehringer Ingelheim Pharma GmbH & Co. KG, Biberach an der Riss, Germany; ‡ Discovery Research, Biotherapeutics Discovery, Boehringer Ingelheim Pharma GmbH & Co. KG, Biberach an der Riss, Germany

## Abstract

Regulatory T cells (Treg) play a critical role in controlling immune responses in diseases such as cancer or autoimmunity. Activated Treg express the membrane protein GARP (LRRC32) in complex with the latent form of the immunosuppressive cytokine TGF-β (L-TGF-β). In this study, we confirmed that active TGF-β was generated from its latent form in an integrin-dependent manner and induced TGF-β receptor signaling in activated human Treg. We studied a series of Abs targeting the L-TGF-β/GARP complex with distinct binding modes. We found that TGF-β receptor signaling could be inhibited by anti–TGF-β and by some, but not all, Abs against the L-TGF-β/GARP complex. Cryogenic electron microscopy structures of three L-TGF-β/GARP complex–targeting Abs revealed their distinct epitopes and allowed us to elucidate how they achieve blockade of TGF-β activation. Three different modes of action were identified, including a novel unusual mechanism of a GARP-binding Ab. However, blockade of GARP or TGF-β by Abs did not influence the suppressive activity of human Treg in vitro. We were also not able to confirm a prominent role of GARP in other functions of human Treg, such as FOXP3 induction and Treg stability. These data show that the GARP/TGF-β axis can be targeted pharmacologically in different ways, but further studies are necessary to understand its complexity and to unleash its therapeutic potential.

## Introduction

Regulatory T cells (Treg) are naturally present in the immune system and play a crucial role in inhibiting several aspects of the immune response (1). They are usually characterized by expression of CD4, the transcription factor FOXP3, and high levels of CD25. Treg are found at high frequencies in tumor tissues of numerous cancer types and may also be found in draining lymph nodes and blood of patients (1). They appear to have a profound effect on antitumor immunity and may represent one important cause of resistance against immunotherapy. In mice, Treg depletion induced tumor immunity, led to tumor growth inhibition, and synergized with immunotherapy in several models (2, 3). In humans, high Treg infiltration was significantly associated with shorter overall survival in the majority of solid tumors, but the prognostic effect varied according to tumor site (1). The clinical benefit of the immune checkpoint blocker anti-CTLA4 may be attributed at least in part to depletion of Treg from tumor tissue (4). Combination of anti-CTLA4 with anti–PD-1 was more effective in the treatment of patients with advanced melanoma than either agent alone (5), potentially indicating a beneficial effect of Treg modulation.

TGF-β is a pleiotropic cytokine that exists in three isoforms (β1, β2, and β3) and has critical functions in the immune system and especially for Treg (6, 7). TGF-β is synthesized as a proprotein that is cleaved in the Golgi apparatus by a furin-like convertase. The resulting latent form of TGF-β (L-TGF-β) is composed of latency-associated peptide (LAP) and mature TGF-β (mTGF-β), which remain noncovalently associated. This complex can further associate with latent TGF-β–binding protein (LTBP) to produce a large latent form for deposition onto the extracellular matrix. Active mTGF-β can be released by interaction of LAP with integrins, including α_v_β_6_ or α_v_β_8_ (8, 9). Cell contraction exerts a physical force that dissipates the complex. Active TGF-β binds to TGF-β receptors, which leads to phosphorylation of SMAD2 and SMAD3.

Alternatively, L-TGF-β can also bind to the cell surface molecule GARP (LRRC32). GARP is highly expressed in activated Treg and platelets and is critical for tethering TGF-β to the cell surface of these cells (10). GARP forms a horseshoe structure consisting of 20 leucine-rich repeats that form an interior parallel β-sheet and an exterior array with a more irregular mix of secondary structure. L-TGF-β is covalently linked to GARP via two disulfide bonds formed between LAP and GARP (11). Tethering of L-TGF-β by GARP occurs on the opposite side of the RGD integrin-binding motifs in LAP. Conversion of L-TGF-β to active TGF-β and release from the L-TGF-β/GARP complex on the surface of Treg is dependent on α_v_β_8_ integrins (12). Structural analysis suggested an alternative activation mechanism in which α_v_β_8_ integrin induced a conformational change in the L-TGF-β/GARP complex, so that TGF-β was able to activate signaling without being released (9, 13). In this activation model, L-TGF-β is expressed in complex with GARP on the cell surface of one cell, gets activated by binding to α_v_β_8_ on another cell, and then exclusively signals to the L-TGF-β–presenting cell. This mechanism may be highly relevant for the tumor microenvironment, as it was found that an α_v_β_8_/L-TGF-β complex formed between α_v_β_8_-expressing tumor cells and L-TGF-β–presenting T cells and was associated with Treg enrichment in tumors (13).

The exact role and relevance of TGF-β for Treg function is still not completely understood and may differ to some extent between mice and humans (14). Nakamura et al. (15) have raised the possibility that TGF-β produced by Treg is bound to their surface and could mediate suppression of T effector cell proliferation in a cell contact–dependent fashion. In their study, suppression could be reversed by high concentrations of anti–TGF-β. In another study, neutralizing Abs against surface-bound TGF-β on Treg blocked the suppression and restored CD8^+^ T cell–mediated killing of tumor cells (16). However, other studies reported conflicting results that high concentrations of anti–TGF-β did not reverse Treg-mediated suppression in vitro (17). Similarly, the impact of GARP on Treg function and stability is not fully clear. GARP-deficient Treg develop normally and are fully competent suppressors of the activation of conventional T cells in vitro (18) and in vivo (19). In contrast, recombinant soluble GARP had potent anti-inflammatory properties in vitro and in vivo (20). Anti-GARP Abs that recognized TGF-β/GARP complexes blocked the production of active TGF-β by a Treg clone and inhibited the immunosuppressive activity of human Treg in vitro and in vivo (21). One of these Abs (clone MHG-8) bound a mixed conformational epitope, concomitantly contacting residues from GARP and L-TGF-β, which may sterically prevent active TGF-β release or the conformational change required for activation. In addition, an Ab against β_8_ integrin blocked TGF-β activation in vitro and inhibited immunosuppression by human Treg in a model of xenogeneic graft-versus-host disease (22).

Blocking formation of active TGF-β from L-TGF-β/GARP may represent a novel mode of action to inhibit Treg function in cancer without depletion. However, when mice with a Treg-specific GARP knockout were challenged orthotopically or s.c. with tumor cells, they did not show prolonged survival or delayed tumor growth compared with wild-type mice (19). Selective blockade of TGF-β1 activation on Treg with an Ab against mouse TGF-β1/GARP complexes did not inhibit tumor growth in mouse tumor models as monotherapy (23). In combination with anti–PD-1, anti–TGF-β1/GARP treatment induced regressions of mouse tumors that were resistant to anti–PD-1 alone. The effects were dependent on CD8^+^ T cells and IFN-γ without depleting Treg within tumors. The relative contributions of TGF-β/GARP expressed on Treg versus platelets is not fully clear (23, 24). An anti-LAP Ab (clone 28G11) was also able to reduce the release of membrane-bound TGF-β from cells. Treatment with such an Ab enhanced antitumor immune responses and reduced tumor growth in several mouse models (25).

In this study, we aimed to explore whether it is possible to influence Treg function by Abs binding to L-TGF-β/GARP. We produced and generated a series of Abs with different binding specificities and analyzed their function and mode of action. We identified Abs that inhibited formation of active TGF-β and associated TGF-β signaling. Biolayer interferometry (BLI) and cryogenic electron microscopy (cryo-EM) studies revealed three different modes of action, confirming one published mechanism and including a novel unusual mechanism of an Ab that binds only to GARP. However, blockade of TGF-β signaling by these Abs did not influence the function of Treg in vitro.

## Materials and Methods

### Abs and reagents

Human TGF-β1 was purchased from R&D Systems. Anti–TGF-β (1D11, R&D Systems) was used for blocking experiments. For FACS analysis, anti-GARP (G14D9, eFluor 660, eBioscience) was used. Recombinantly expressed anti–L-TGF-β/GARP or isotype control Abs were labeled using a Lightning-Link rapid Alexa Fluor 488 Ab labeling kit (Novus Biologicals).

### Recombinant expression and purification of Abs

For Abs reported in literature or patents, DNA encoding for the H and L chain of Abs was codon optimized for expression in human cell lines, synthesized (GeneArt, Thermo Fisher Scientific), and cloned into mammalian expression vectors. HEK293-E6 cells were transiently transfected with expression vectors for the H and L chains, and 1% tryptone N1 feed was added after 24 h. Expression was stopped after a total of 5 d and cells were removed by centrifugation. Abs were purified from the supernatant by affinity chromatography using protein A MabSelect SuRe beads (GE Healthcare). Purified Abs were stored in PBS. Integrity of the Abs was analyzed by SDS-PAGE and analytical size-exclusion chromatography. Mouse Abs from an internal immunization campaign were purified from hybridoma supernatants.

To generate Fab fragments, full-length Abs were cleaved using papain immobilized on 6% beaded agarose (Pierce). The Abs were concentrated to 4 mg/ml with Amicon Ultra-15 centrifugal filter units (EMD Millipore), molecular mass cutoff of 100 kDa, and during concentration the buffer was replaced by papain digestion buffer. One hundred twenty-five microliters of papain slurry per microgram of Ab was equilibrated in papain digestion buffer as well. Abs and papain were mixed, aliquoted into 2-ml reaction tubes (Eppendorf), and placed in a shaker incubator at 37°C for 5 h. After complete cleavage of the full-length Abs, the papain beads were removed by gravity flow through an empty PD-10 column (GE Healthcare Life Sciences). The Fc fragment was removed by affinity chromatography using protein A MabSelect SuRe beads (GE Healthcare).

### Recombinant expression and purification of GARP, GARP/L-TGF-β, and L-TGF-β protein

DNA encoding for GARP extracellular domain (aa 20–627) fused to the Fc sequence of a human IgG1 and a C-terminal His tag (GARP-Fc-His), L-TGF-β1 (aa 30–390), and His-L-TGF-β1 (N-terminal His tag followed by aa 30–390 of L-TGF-β1) was codon optimized for expression in human cell lines, synthesized (GeneArt, Thermo Fisher Scientific), and cloned into pOptiVEC (GARP-Fc-His) and pcDNA3.1 (L-TGF-β1) expression vectors, respectively. HEK293-E6 cells were transiently transfected, and 1% tryptone N1 feed was added after 24 h. Expression was stopped after a total of 5 d and cells were removed by centrifugation. Recombinant GARP-Fc-His, GARP-Fc-His/L-TGF-β, and His/L-TGF-β were purified from the supernatant by affinity chromatography using Ni-NTA beads (GE Healthcare) followed by size-exclusion chromatography. The Fc-His part of both GARP constructs was subsequently cleaved using papain immobilized on 6% beaded agarose (Pierce), as described above. The Fc-His fragment was removed by affinity chromatography using protein A MabSelect SuRe beads (GE Healthcare). Purified proteins were stored in PBS. Integrity of the individual proteins and the complex was analyzed by SDS-PAGE and analytical size-exclusion chromatography.

### Binding characterization of Abs in Octet and surface plasmon resonance

To understand the binding specificity of our set of Abs, their interaction with either GARP, GARP/L-TGF-β, or L-TGF-β was analyzed by BLI. All steps were performed in PBS buffer. Murine and human Abs with a concentration of 10 µg/ml were captured for 180 s on AMC (anti-murine IgG Fc capture) and AHC (anti-human IgG Fc capture) tips, respectively. Binding to 10 µg/ml GARP, GARP/L-TGF-β, or L-TGF-β was then analyzed by incubation with the Ab-loaded tips for 180 s, followed by a 600-s dissociation phase.

### Cryo-EM sample preparation and data collection

The ternary complexes containing GARP/L-TGF-β and the respective Fab molecules (LHG-10, 28G11, or 12G2B4) were formed by incubation for 30 min at 4°C with an excess of Fab. The complexes were purified using size-exclusion chromatography (Superdex 200 10/300 column [GE Healthcare]) in 15 mM HEPES (pH 7.5), 100 mM NaCl. For cryo-EM grid preparation, 3 μl of the respective Fab-GARP/L-TGF-β at 0.4 mg/ml was applied to glow-discharged Quantifoil R1.2/1.3 300-mesh gold grids. The sample was then vitrified with a Leica EM GP at 80% humidity using 2-s blotting time. Cryo-EM data were acquired at 300 kV on a FEI Titan Krios. Dose-fractionated video frames with a physical pixel size of 1.08 or 0.83 Å were recorded on a K2 summit or Gatan K3 direct electron detector applying a defocus range varying from 2.4 to 0.8 Å. A 1-s exposure with 33–40 subframes was recorded in the counting mode with a total dose of 39–45 electrons/Å^2^. Supplemental Table I shows a summary of all relevant information on the cryo-EM data collection and model validation parameters.

### Cryo-EM data processing and model building

The collected cryo-EM videos were dose-weighted and corrected for beam-induced motion using MotionCorr (26). Contrast transfer function estimation and further processing steps were done using the CryoSPARC software package (27). Particles were picked using a template-based approach in CryoSPARC. The particles were extracted using a box size of 300 × 300 pixels and processed in several successive two-dimensional classification rounds. Ab initio three-dimensional models were created using CryoSPARC, and the particle stack was further unified by three-dimensional classification. The final three-dimensional reconstruction was calculated using the nonuniform refinement tool in CryoSPARC (28).

The crystal structure of the MHG-8-GARP/L-TGF-β complex (PDB: 6GFF) (11) was used as an initial model and fitted into the cryo-EM map using the Phenix suite (29). The LHG-10-GARP/L-TGF-β map quality was further improved using density modification (30). The LHG-10-GARP/L-TGF-β structure was built in iterative cycles of manual adjustment in COOT (31) and real-space refinement using Phenix.real_space_refine (32). The refinement process was monitored with MolProbity (33). Structure figures were generated using PyMOL (Schrödinger) (34) and UCSF Chimera (35). Chimera was used to dock the GARP/L-TGF-β complex and MHG-8 separately (PDB: 6GFF) into the low-resolution maps of the two other Fab complexes. Supplemental Table I shows a summary of relevant information on the cryo-EM data collection and model validation parameters.

The cryo-EM maps of all Fab complexes have been deposited in the Electron Microscopy Data Bank (https://www.ebi.ac.uk/emdb/) with access numbers EMD-16460, EMD-16456, and EMD-16459 The coordinates of the LHG-10-GARP/L-TGF-β complex have been deposited to the Protein Data Bank (https://www.rcsb.org/) with access code 8C7H.

### Purification of immune cells

Buffy coats of healthy blood donors were received from the Institute of Clinical Transfusion Medicine, Ulm, Germany. All experiments were approved and followed ethical standards and local regulations. PBMC were purified by Ficoll gradient centrifugation. Treg (CD4^+^CD25^+^) and conventional T cells (Tcon; CD4^+^CD25^−^) were isolated by a human CD4^+^CD25^+^ Treg isolation kit (Miltenyi Biotec) according to the manufacturer’s protocol. Briefly, CD4^+^ T cells were isolated by negative selection, followed by positive selection of CD25^+^ cells (Treg). The remaining CD4^+^CD25^−^ fraction was used as Tcon. Allogeneic APC were prepared by depletion of CD3^+^ cells from PBMC of a different donor using CD3 microbeads (Miltenyi Biotec). For some experiments, untouched CD3^+^ pan-T cells were isolated (EasySep human T cell isolation kit, STEMCELL Technologies).

### p-Smad assay

Treg or Tcon were stimulated in 96-well round-bottom plates (50,000 cells/well) in serum-free TexMACS medium (Miltenyi Biotec) with anti-CD3/CD28 human T activator Dynabeads (Life Technologies) at a 1:1 ratio. The indicated Abs or reagents were added at the same time as the stimulus. For testing dependence on integrins, an RGD peptide (GRRGDLATIH) was used or an RGE peptide (GRRGELATIH) as control (22). As a positive control active TGF-β (10 ng/ml) was added where indicated. Three days after stimulation, p-Smad3 was quantified using a p-SMAD3 (Ser^423/425^) AlphaLISA assay (PerkinElmer) according to the manufacturer’s instructions. Where indicated, luminescence values were normalized to the signal of stimulated cells without inhibitor for each donor individually.

### Treg suppression assay and FOXP3 induction

Tcon were labeled with CellTrace Violet (Molecular Probes) and seeded into 96-well round-bottom plates (50,000 cells/well). Treg from the same donor were added at different ratios as indicated in the figures. T cells were stimulated with allogeneic APC (50,000 cells/well) and anti-CD3 (OKT3, eBioscience; 0.5 μg/ml) in the absence or presence of the indicated Abs. After 5 d, cells in the coculture were stained with anti-CD3 (SK7, Becton Dickinson) and analyzed by FACS (Fortessa X20, Becton Dickinson). For measuring proliferation of Tcon by CellTrace Violet dilution, gating on CD3^+^ CellTrace Violet^+^ cells was performed, and the percentage of CellTrace Violet^low^ cells was determined. Data were normalized to the proliferation of Tcon stimulated in the absence of Treg for each donor individually. To determine FOXP3 induction in Tcon, cells in the coculture were permeabilized (human FOXP3 buffer set, Becton Dickinson), stained with anti FOXP3 (259D/C7, Becton Dickinson), and analyzed by FACS. Tcon were identified by gating on CD3^+^ CellTrace Violet^+^ cells, and the percentage of FOXP3^+^ Tcon was determined. In some experiments, Treg were preactivated with allogeneic APC and anti-CD3 in 96-well round-bottom plates. After 24 h, CellTrace Violet–labeled Tcon (50 000 cells/well) from the same donor as the Treg were added. After 5 d analysis was performed as above.

### Treg stability

To test the stability of Treg, CD4^+^CD25^+^ T cells were incubated in 96-well round-bottom plates (10^6^ cells/well) in TexMACS medium (Miltenyi Biotec) with 5% heat-inactivated human serum and stimulated with CD3/CD28 MACSiBead particles (Miltenyi Biotec; cell/bead ratio 1:4) and IL-2 (500 U/ml). Abs or TGF-β was added as indicated in the figures. After 5 d, cells were first stained with fixable viability stain (Becton Dickinson) and then fixed, permeabilized, and stained for FOXP3. The percentage of FOXP3^+^ cells in the viable fraction was determined by FACS. To generate induced Treg, CD4^+^CD25^−^ T cells were isolated and stimulated with allogeneic APC and anti-CD3 in the presence of TGF-β (2 ng/ml). After 5 d, CD3^+^ cells were purified from the coculture using a pan-T cell isolation kit (human) (Miltenyi Biotec) and resuspended in fresh medium containing IL-2 and the indicated Abs or TGF-β. Three days later the percentage of FOXP3^+^ cells was determined as above.

## Results

### Production and characterization of GARP protein and Abs

To investigate the complex biology of GARP and L-TGF-β, these proteins as well as the corresponding L-TGF-β/GARP complex were produced recombinantly. To analyze the effects of pharmacological intervention with TGF-β/GARP biology we produced a set of tool Abs that have either been reported before or were the result of an internal mouse immunization campaign (Table I). BLI analysis was used to understand the binding specificity and thereby give an initial hint about the probable epitope of the respective Abs. Interestingly, we identified molecules that selectively bound either to GARP or L-TGF-β, respectively (Table I). These Abs bound both to the individual proteins and to the L-TGF-β/GARP complex. In contrast, Abs MHG-8 and LHG-10 bound to the L-TGF-β/GARP complex only, but not to the individual proteins, as published previously (21).

**Table I.** Binding specificity and functional activity of tool Abs

mAb	Binding to			Inhibition of SMAD3 phosphorylation	Ref.
	L-TGF-β	GARP	L-TGF-β/GARP		
28G11	√	X	√	√	(44)
AB2	√	X	√	X	(45)
MHG-8	X	X	√	√	(21)
LHG-10	X	X	√	√	(21)
105F	X	√	√	X	(46)
110F	X	√	√	X	(46)
5C5	X	√	√	(√)	(47)
3C9	X	√	√	(√)	—
15A3	X	√	√	X	—
21A11	X	√	√	X	—
12A2F8	X	√	√	√	—
12B9H5	X	√	√	√	—
12G2B4	X	√	√	√	—
14G2D8	X	√	√	X	—
21A11B7	X	√	√	X	—
22H6H5	X	√	√	X	—

√, binding or inhibiting, respectively; X, no binding or no inhibition, respectively; (√), partial inhibition; —, from internal campaign.

### Cryo-EM structure reveals mode of action of anti-GARP Abs

To understand the distinct modes of action of the Abs in molecular detail, it is advantageous to know the binding epitope of the Ab. Therefore, we analyzed the Fab-GARP/L-TGF-β complex structures of three Fab molecules, which differentially recognized the GARP, L-TGF-β, and the GARP/L-TGF-β complex in the BLI experiment, using single-particle cryo-EM. For this purpose, we generated Fab fragments from the recombinant Abs 28G11, LHG-10, and 12G2B4 and purified complexes of the Fabs with recombinant GARP/L-TGF-β via size-exclusion chromatography. The protein complexes were placed on EM grids and plunge frozen in liquid ethane. Roughly 3000 videos of each Fab-GARP/L-TGF-β complex were recorded using a FEI Titan Krios microscope.

We were able to successfully reconstruct maps of the three Fab-GARP/L-TGF-β complexes at different resolutions. The maps of 12G2B4 and 28G11 offer details up to a resolution of 3.4 and 7.5 Å, respectively. The 12G2B4 map suffered from a strong orientation bias of the particle stack, which limited the reconstruction resolution. In these maps, the models of the crystal structure 6GFF (GARP/L-TGF-β complex and MHG-8) were separately docked by rigid body refinement and fitted using Chimera (Supplemental Fig. 1A–D). The reconstitution of the LHG-10 complex, in contrast, led to a map with 2.7 Å resolution, which allowed us to derive an atomic structure of the complex (Supplemental Fig. 1E, 1F).

**FIGURE 1.**
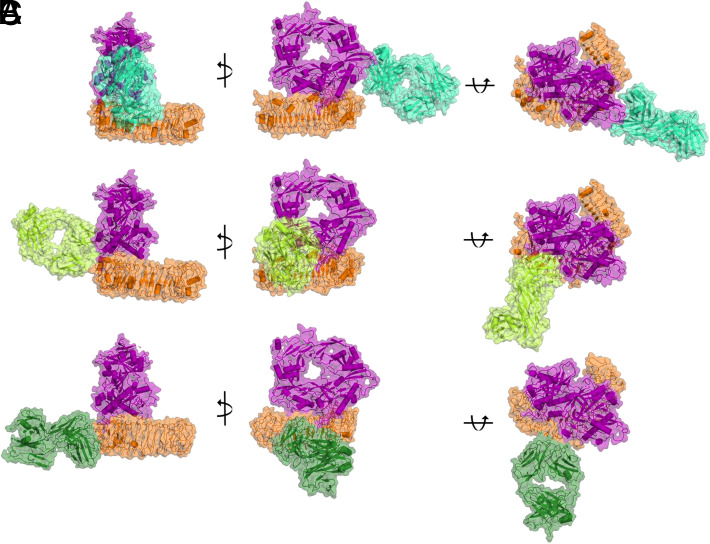
Cryo-EM structures depict binding epitopes of three neutralizing Fabs on the GARP/L-TGF-β complex. Cartoon and surface representations show cryo-EM GARP/L-TGF-β-Fab models from different angles. GARP is depicted in orange and L-TGF-β in magenta. (**A**) Fab 28G11 is depicted in cyan, (**B**) LHG-10 in lemon green, and (**C**) 12G2B4 in dark green.

In Fig. 1 the three distinct ternary complexes are depicted showing that the respective Fabs bind to distinct epitopes of the GARP/L-TGF-β complex. 28G11 (Fig. 1A) interacts exclusively with L-TGF-β. This interaction is mediated by loop regions 301–313 and 370–374 of mTGF-β and loop 60–72 of LAP. The second identical epitope of the 2-fold symmetric L-TGF-β is not available for Fab binding due to a steric block by the unsymmetrically bound GARP such that a complex of 28G11/GARP/L-TGF-β with 1:1:1 stoichiometry is formed.

LHG-10 recognizes the GARP/L-TGF-β complex by binding to the interface of GARP and L-TGF-β, similarly to and in close proximity to the binding epitope of MHG-8 (11) (Fig. 1B). The epitope consists of several residues on LAP (residues 269–273, 58, 100, 104), mTGF-β (residues 336–338, 345), and GARP (residues 137, 140–143, 161–163, 165, 167) (Supplemental Fig. 2).

Fig. 1C depicts the binding mode of 12G2B4, which recognizes the GARP/L-TGF-β complex by direct interaction with GARP residues 113–124, 139–149, 165–173, 189–220, 213–220, and 236–243. At this position on the GARP surface its horseshoe fold is complemented by a β-strand extension contributed by LAP (Supplemental Fig. 3A). It is very likely that there is no direct interaction between 12G2B4 and the intercalating LAP N-terminus, and solely the adjacent GARP residues are recognized. However, because the Fab sequence was not recovered and the resolution is limited, a more precise analysis is not feasible. Remarkably, 12G2B4 induces a complex conformational change in GARP. Although in the LHG-10 and MHG-8 bound structure or in the GARP/L-TGF-β/α_v_β_8_ integrin structure (7Y1R), GARP possesses a very similar conformation, it adopts a different conformational in the 12G2B4 costructure. In the 12G2B4 bound state the horseshoe fold of GARP is bent to a larger extent and GARP twists toward the L-TGF-β complex (Supplemental Fig. 3B–E). At the position at which GARP shows the largest conformational change compared with the LHG-10 bound state, the backbone of, for example, Arg446 shifts by 6.6 Å.

### Production of active TGF-β by activated Treg is dependent on integrins and GARP

**FIGURE 2.**
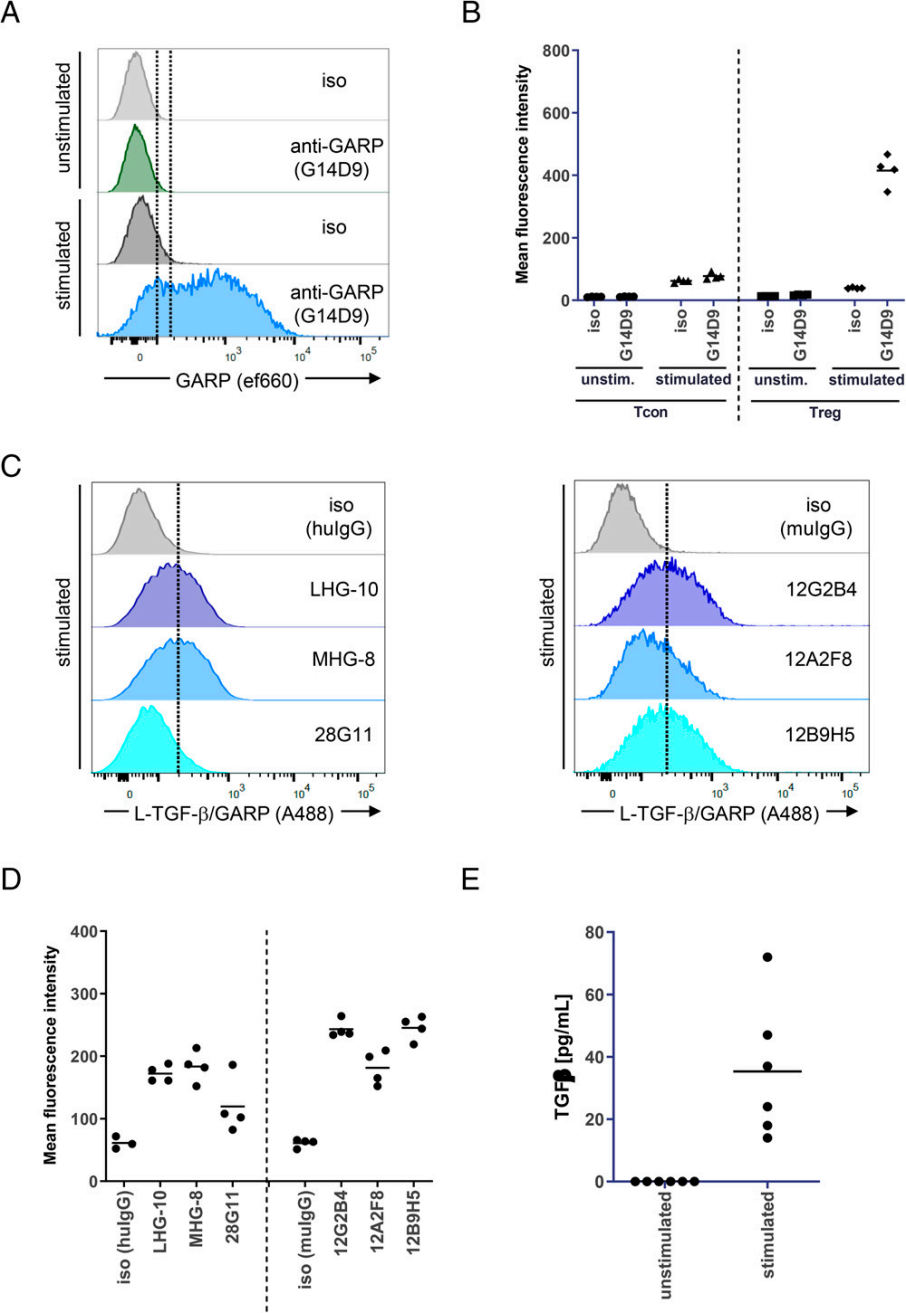
Anti–L-TGF-β/GARP Abs bind to GARP expressed on activated Treg. (**A**) Activated Treg express GARP. CD4^+^CD25^+^ T cells (Treg) were isolated from buffy coats and were activated with anti-CD3/CD28 beads for 3 d. GARP expression was determined by FACS directly after isolation (unstimulated) and after activation (stimulated) using the commercial anti-GARP AbG14D9. Histograms from one representative donor are shown. (**B**) Aggregate mean fluorescence intensity (MFI) data from four donors from the experiment described in (A) (Treg). In addition, Tcon from the same four donors were isolated, activated, and tested for GARP expression. (**C**) Anti–L-TGF-β/GARP Abs bind to activated Treg. Treg were isolated and activated as described in (A). Binding of the indicated anti–L-TGF-β/GARP Abs to activated Treg was determined by FACS. In the graph, histograms from the same donor as in (A) are shown and were grouped according to the Ab Fc part (left, human IgG; right, murine IgG). (**D**) Aggregate MFI data from four donors from the experiment described in (C). The experiment was done in parallel to (A) and (B) using the same donors. (**E**) Stimulated Treg release small amounts of TGF-β. CD4^+^CD25^+^ T cells were activated with anti-CD3/CD28 beads in serum-free medium for 3 d or left untreated. The concentration of TGF-β in supernatants was measured by ELISA (*n* = 6 donors).

We aimed to explore whether it is possible to influence Treg function by a diverse set of Abs with distinct binding modes to GARP/L-TGF-β. Therefore, we first determined expression of GARP on Treg. We stimulated CD4^+^CD25^+^ Treg with anti-CD3/CD28 and could confirm GARP expression by FACS on activated, but not on unstimulated, Treg (Fig. 2A). Neither stimulated nor unstimulated Tcon expressed GARP (Fig. 2B). Furthermore, we could confirm that different L-TGF-β/GARP Abs bound to stimulated Treg (Fig. 2C, 2D), demonstrating that their target was present. However, the staining intensity of the anti-LAP Ab 28G11 was only weak. None of these Abs bound to unstimulated Treg or Tcon, in line with lack of GARP expression as determined above (data not shown).

**FIGURE 3.**
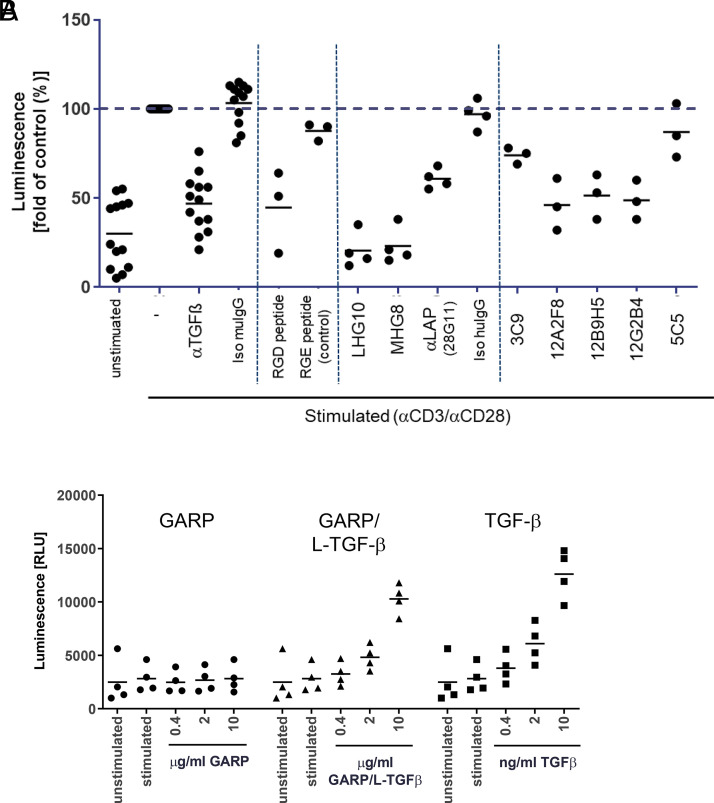
Generation of active TGF-β by stimulated Treg is integrin-dependent and can be inhibited by anti-GARP Abs. (**A**) Inhibition of TGF-β–dependent signaling. CD4^+^CD25^+^ T cells were left untreated (unstimulated) or were activated with anti-CD3/CD28 beads for 3 d (stimulated) in the presence of the indicated Abs (10 μg/ml) or peptides (230 μM). Smad3 phosphorylation was quantified using an AlphaLISA kit. Data were normalized to the signal of stimulated cells without inhibitor for each donor individually. In the graph four separate experiments with 13 donors in total were summarized; each condition was run with at least 3 donors. (**B**) Induction of Smad3 phosphorylation in pan T cells. CD3^+^ pan T cells were left untreated (unstimulated) or were activated with anti-CD3/CD28 beads for 3 d (stimulated) in the presence of the indicated reagents. Smad3 phosphorylation was quantified using an AlphaLISA kit (*n* = 4 donors).

Next, we analyzed whether TGF-β was released by Treg. Stimulated Treg released only small amounts of TGF-β into the supernatant (Fig. 2E). To determine whether Treg expressed active TGF-β that was able to induce TGF-β receptor signaling, we quantified Smad3 phosphorylation in Treg using an AlphaLISA kit (Fig. 3A). In unstimulated Treg only low background levels of p-Smad3 were detected. Stimulation by anti-CD3/CD28 led to an increase of p-Smad3 by a factor of 2–20, depending on the donor (average 3.3-fold increase). Smad3 phosphorylation could be inhibited by addition of anti–TGF-β (Fig. 3A). Interestingly, an RGD peptide that blocks interaction with integrins, but not a control RGE peptide (22), also inhibited Smad3 phosphorylation, confirming an essential role of integrins in the activation of TGF-β from the GARP complex (Fig. 3A).

In addition, we tested the ability of different L-TGF-β/GARP–binding Abs to inhibit active TGF-β formation (Fig. 3A, Table I). We identified several Abs against the L-TGF-β/GARP complex that reduced Smad3 phosphorylation, including 28G11 (anti–L-TGF-β), 12G2B4 (anti-GARP), and LHG-10 (anti–L-TGF-β/GARP). However, other Abs with similar binding specificity in BLI did not show a functional effect (Table I).

Taken together, we confirmed that active TGF-β was generated from its latent form in an integrin-dependent manner and induced TGF-β receptor signaling in activated Treg and that TGF-β receptor signaling could be inhibited by anti–TGF-β and by some, but not all, Abs targeting the L-TGF-β/GARP complex.

Furthermore, we analyzed Smad3 phosphorylation in CD3^+^ pan T cells (containing CD8^+^ and CD4^+^ T cells, but only negligible amounts of Treg). In contrast to Treg, stimulation of pan T cells did not increase p-Smad3 levels (Fig. 3B). Recombinant active TGF-β or the recombinant L-TGF-β/GARP complex induced Smad3 phosphorylation in stimulated T cells. L-TGF-β/GARP–induced p-Smad3 could be inhibited by anti–TGF-β or anti–L-TGF-β (28G11) (data not shown). Purified recombinant “empty” GARP (without L-TGF-β) did not induce Smad3 phosphorylation.

### Suppression of effector T cell proliferation by Treg is not influenced by anti–TGF-β or anti-GARP Abs

To estimate the extent of immunosuppression by TGF-β in vitro, we stimulated T cells with plate-bound anti-CD3 for 5 d and measured their proliferation in the presence or absence of different concentrations of active TGF-β (data not shown). At the maximum concentration used (10 ng/ml) TGF-β inhibited proliferation by 32%. Similarly, L-TGF-β/GARP inhibited proliferation of anti-CD3–stimulated T cells (data not shown). Immunosuppression by TGF-β or the recombinant L-TGF-β/GARP complex was also measured in MLR. Both reagents strongly inhibited IFN-γ secretion in a dose-dependent manner (data not shown).

**FIGURE 4.**
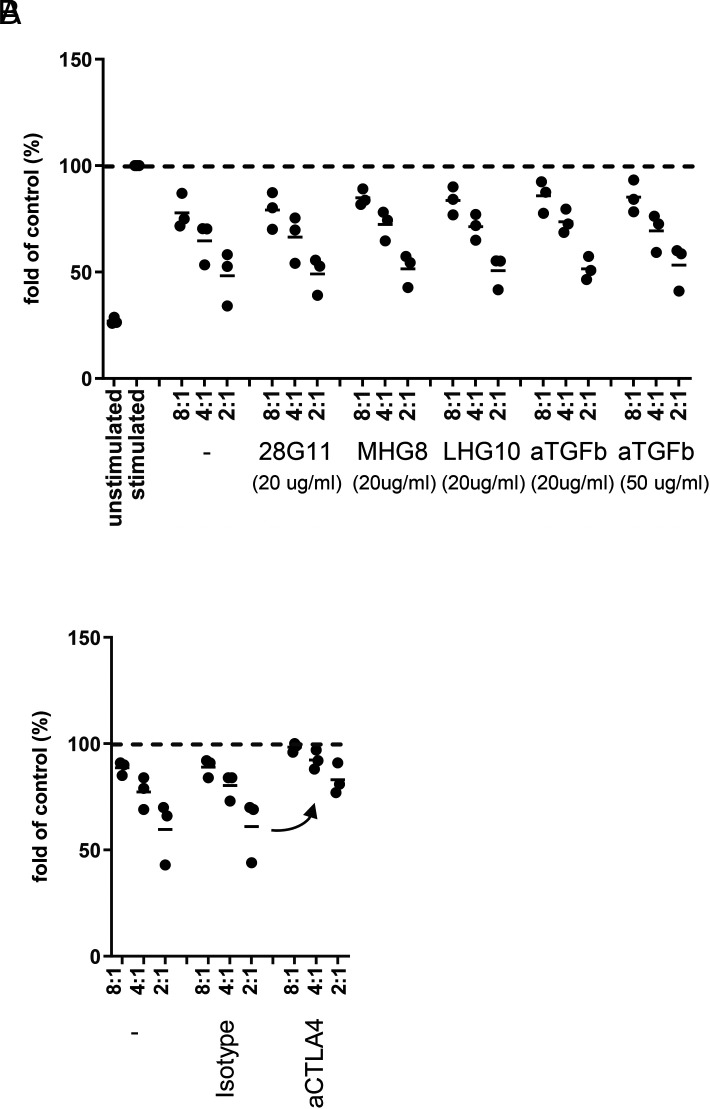
Suppression of Tcon proliferation by Treg is not influenced by anti–TGF-β or anti-GARP Abs. (**A**) Tcon were labeled with CellTrace Violet and seeded alone or with Treg at different Tcon/Treg ratios (8:1, 4:1, 2:1). Cells were stimulated with allogeneic APC and anti-CD3 (0.5 μg/ml) in the absence or presence of the indicated Abs. Proliferation of Tcon was measured by CellTrace Violet dilution after 5 d. Data were normalized to the proliferation of stimulated Tcon alone for each donor individually (*n* = 3 donors). (**B**) As a positive control, anti-CTLA4 (20 μg/ml) or an isotype control was tested with three separate donors.

To investigate the role of TGF-β or GARP on Treg function, we tested various blocking Abs in a Treg suppression assay (Fig. 4). Human Tcon were seeded alone or with different numbers of Treg and were stimulated with allogeneic APC and anti-CD3. Treg inhibited proliferation of Tcon up to ∼50% at the highest Treg cell number used. In this assay, neither an anti–TGF-β Ab, nor the anti-LAP Ab (28G11), nor the published anti–L-TGF-β/GARP Abs MHG-8 and LHG-8 had any influence on Tcon proliferation (Fig. 4A). Also, the anti-GARP Abs that were active in the Smad3 phosphorylation assay (Fig. 3A) did not reverse Tcon proliferation (data not shown). In contrast, anti-CTLA4 was able to increase Tcon proliferation (Fig. 4B).

Taken together, consistent with literature reports (7, 14), but in contrast to others (21), we could not identify a key role for TGF-β in suppression of T cell proliferation by Treg in vitro. Accordingly, in our experiments anti-GARP or anti–GARP/L-TGF-β Abs that block the formation of active TGF-β were not able to influence the suppressive effect of Treg.

### Active TGF-β, but not Treg, enhances FOXP3 expression in Tcon

Conventional mouse T cells converted to FOXP3^+^ Treg following in vitro stimulation in the presence of TGF-β (14, 36). Such induced Treg (iTreg) could represent a mechanism of “infectious tolerance” in which Treg educated naive T cells to become suppressive cells. In humans, stimulation of CD4^+^ T cells under similar conditions also resulted in the expression of FOXP3, but the cells may have lacked regulatory cell function (14, 36). Induction of FOXP3 expression in naive human T cells cocultured with Treg was decreased when Treg were transfected with a small interfering RNA targeting TGF-β or GARP (10).

**FIGURE 5.**
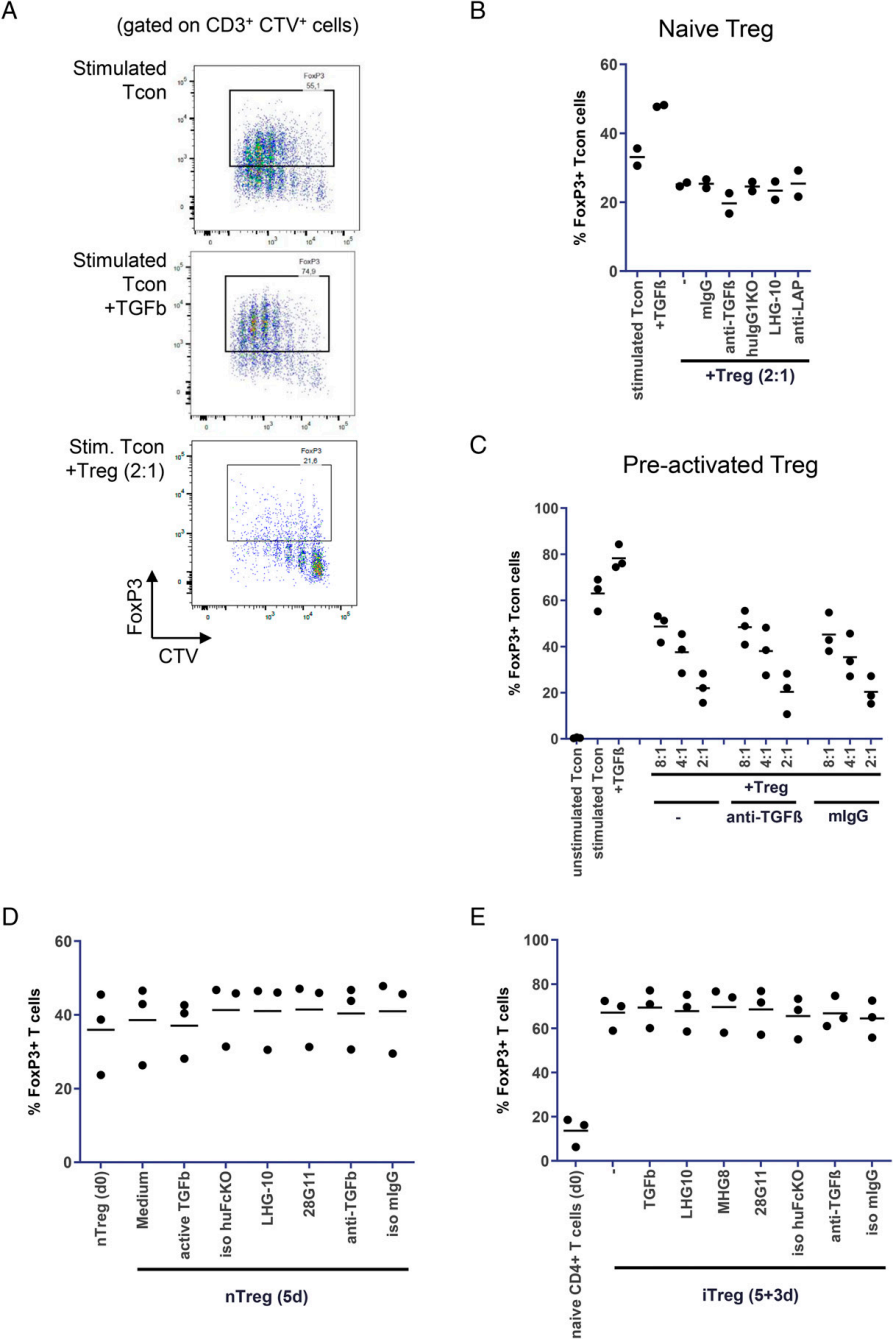
Treg do not enhance FOXP3 in Tcon, and neither TGF-β nor GARP enhances stability of Treg. (**A**) No FOXP3 induction in Tcon by naive Treg. Similar to Fig. 3, Tcon were labeled with CellTrace Violet and seeded alone or with naive Treg (Tcon/Treg ratio 2:1). Cells were stimulated with allogeneic APC and anti-CD3. In a control sample active TGF-β (2 ng/ml) was added in the absence of Treg. After 5 d cells were stained for FOXP3 and analyzed by FACS. Tcon were identified by gating on CD3^+^ CellTrace Violet^+^ (CTV^+^) cells, and the percentage of FOXP3^+^ Tcon was determined as shown in exemplary dot plots from one representative donor. Aggregate data from multiple donors are plotted in (**B**) and (**C**). (B) No influence of Abs on FOXP3 levels in Tcon. As in (A), Tcon were stimulated and cocultured with naive Treg. The indicated Abs (20 μg/ml) were added prior to stimulation and coculture. Percentage of FOXP3^+^ Tcon was determined as in (A) (*n* = 2 donors). (C) Reduction of FOXP3 induction in Tcon by preactivated Treg. Treg isolated from buffy coats were stimulated with allogeneic APC and anti-CD3. After 24 h, anti– TGF-β Ab (20 μg/ml) or isotype control was added. One hour later, CellTrace Violet–labeled Tcon from the same donor as the Treg were added (Tcon/Treg ratios 8:1, 4:1, 2:1). After 5 d, analysis was performed as in (A) and (B). (**D**) Stability of nTreg. CD4^+^CD25^+^ T cells were isolated from buffy coats and stained for FOXP3 expression (nTreg d0). Unstained nTreg were incubated with anti-CD3/CD28 beads (1:4) and IL-2 (500 U/ml) in the absence or presence of the indicated Abs (20 μg/ml) or TGF-β (2 ng/ml). After 5 d cells were stained for FOXP3 and the percentage of FOXP3^+^ cells was determined by FACS (*n* = 3 donors). (**E**) Stability of iTreg. CD4^+^ CD25^-^ T cells were isolated and stained for FOXP3 expression (naive CD4^+^ T cells day 0). The remaining unstained CD4^+^CD25^−^ cells were stimulated with allogeneic APC and anti-CD3 in the presence of TGF-β (2 ng/ml) to induce iTreg. After 5 d, CD3^+^ cells were purified from the coculture and resuspended in fresh medium containing IL-2 and the indicated Abs (10 μg/ml) or TGF-β. Three days later, cells were stained for FOXP3 and the percentage of FOXP3^+^ cells was determined by FACS (*n* = 3 donors).

To study the role of GARP in this process, we stimulated CD4^+^CD25^−^ Tcon with allogeneic APC and soluble anti-CD3 and determined the FOXP3 expression in Tcon (Fig. 5). Stimulation (in the absence of exogenous TGF-β or Treg) induced FOXP3 in Tcon, mainly in cells that had divided (CellTrace Violet^low^ cells; Fig. 5A). Addition of TGF-β (2 ng/ml) did not inhibit proliferation, but it further increased FOXP3 expression in these cells. Coculture of Tcon with naive Treg inhibited proliferation of Tcon as before (Fig 5A, lower panel). Accordingly, the fraction of cells that had divided as well as the percentage of FOXP3^+^ Tcon was reduced (Fig. 5A, 5B). Addition of anti–TGF-β, anti–GARP/L-TGF-β, or anti-LAP Ab did not influence proliferation or FOXP3 expression in Tcon (Fig. 5B and data not shown).

As Treg upregulate GARP after activation, we also preactivated Treg for 24 h before using them in the coculture with Tcon. Preactivated Treg were highly suppressive and strongly inhibited proliferation of Tcon (data not shown). In line with a lower number of divided Tcon, the percentage of FOXP3^+^ Tcon was decreased (Fig. 5C). Again, anti–TGF-β did not have an effect on proliferation or FOXP3 expression.

These results indicate that expression of FOXP3 in human CD4^+^ Tcon correlates with their activation and division and that exogenous TGF-β, but not Treg, can further enhance the expression.

### Neither TGF-β nor GARP enhances short-term stability of Treg

Apart from acting on Tcon, Treg-derived TGF-β may be important to increase the stability of Treg and sustain their expression of FOXP3 (37, 38). To investigate whether TGF-β and GARP have an influence on the stability of Treg, we isolated natural Treg (nTreg) from healthy donors, cultured them for 5 d, and determined their expression of FOXP3 (Fig. 5D). In the three donors used, 36 ± 11% of Treg were FOXP3^+^ directly after isolation. The percentage remained stable during the cultivation period in medium with IL-2. Addition of active TGF-β, or block of GARP, TGF-β, or LAP by Abs, did not change the expression. Viability of Treg was similar in all samples.

As iTreg require TGF-β for their induction, they might be more sensitive to its withdrawal or modulation. Therefore, we stimulated CD4^+^CD25^−^ T cells in the presence of TGF-β to generate FOXP3-expressing model iTreg (Fig. 5E). After 5 d these T cells were purified from the stimulation culture and were resuspended in fresh medium containing IL-2, but no TGF-β. Another 3 d later FOXP3 expression was determined, and 67 ± 7% of these model iTreg expressed FOXP3, indicating that FOXP3 induction by TGF-β was successful and remained high even in the absence of TGF-β. Addition of TGF-β in the 3-d resting period did not further enhance FOXP3 expression. The number of FOXP3^+^ cells was also not changed by the presence of Abs against TGF-β, GARP/L-TGF-β (MHG-8, LHG-10), or LAP (28G11) during the resting period.

In summary, we were not able to confirm a prominent effect of Treg-derived TGF-β, and hence GARP, on Treg stability in our assays.

## Discussion

Treg play a critical role in controlling the antitumor immune response and may represent a cause of resistance against immunotherapy. Activated Treg express the membrane protein GARP in complex with the latent form of the immunosuppressive cytokine TGF-β. Blocking active TGF-β formation from L-TGF-β/GARP may represent a novel mode of action to inhibit Treg function in cancer without depletion of Treg and its associated side effects. Therefore, we aimed to explore whether it is possible to influence Treg function by Abs binding to L-TGF-β/GARP.

Two different models for the activation of TGF-β have been suggested. One model requires actin-cytoskeletal force and the release and diffusion of mTGF-β from the L-TGF-β/GARP complex (39, 40). The other model does not require release and diffusion of TGF-β, but functions through α_v_β_8_-mediated TGF-β activation in a large multicomponent cell–cell protein complex (9).

We produced and characterized a set of tool Abs that either represented Abs that have been reported before or were the result of an internal mouse immunization campaign. These Abs have been raised against different Ags (see references in Table I) and exhibited different binding specificities. Although all Abs bound to the L-TGF-β/GARP complex, only some inhibited TGF-β receptor signaling as quantified by Smad3 phosphorylation. Functional activity was independent of the binding specificity as determined by BLI (Table I).

Cryo-EM structures of Fab fragments binding to the L-TGF-β/GARP complex revealed three different modes of action of the blocking Abs.

Fab 28G11 recognizes an epitope on L-TGF-β that is distinct from the epitope on LAP to which the α_v_β_8_ integrin binds. We assume that by stabilizing the LAP-mTGF-β complex, 28G11 inhibits the release of TGF-β. This stabilization of the GARP/L-TGF-β complex and the related prevention of mTGF-β release, results in the inhibition of p-SMAD3 formation as shown in the p-SMAD3 assay.

BLI data indicate that LHG-10 recognizes solely the GARP/L-TGF-β complex and not the individual protein components. The cryo-EM structure reveals that LHG-10 bridges both proteins and stabilizes the GARP/L-TGF-β complex by binding to the interface of GARP and L-TGF-β. This mode of action, which is identical to the one of MHG-8, results in reduced mTGF-β formation and the inhibition of p-SMAD3 formation as shown in the p-SMAD3 assay.

12G2B4 binds exactly at the point where the horseshoe fold of GARP is extended by a β-strand addition of LAP. In this study, the β-sheet at the N terminus of LAP intercalates into the β-sandwich of GARP. Furthermore, and remarkably, upon binding of 12G2B4 a conformational change of GARP is induced, which comprises a shrinking of the radius of the horseshoe structural motif. We were unable to reconstruct the full atomic details of these conformational changes due to the poor local resolution at the GARP/L-TGF-β portion of the complex. BLI binding data showed binding of GARP alone as well as the GARP/L-TGF-β complex. Binding to GARP alone, however, would not explain the observed behavior of this Ab.

From the structural data, two potential models for the observed inhibition of the mTGF-β formation and of the p-SMAD3 formation can be formulated. 12G2B4 could potentially increase the overall stability of the GARP/L-TGF-β complex by conformationally rearranging GARP and stabilizing GARP in the region where the N-terminal β-sheet of LAP intercalates into GARP. With this potential mode of action 12G2B4 could prevent TGF-β maturation by indirect stabilization of the GARP/L-TGF-β complex, thereby hindering mTGF-β from diffusing. Alternatively, the conformational rearrangement of GARP upon binding of 12G2B4 could have an influence on L-TGF-β conformation and thereby could decrease the accessibility of TGF-β for the TGF-β receptor.

We could confirm that recombinant TGF-β or the recombinant L-TGF-β/GARP complex had immunosuppressive effects on T cells, as demonstrated by induction of Smad3 phosphorylation, reduction of T cell proliferation, and inhibition of IFN-γ secretion in MLR. Stimulation of Treg strongly induced GARP expression and made them highly immunosuppressive. In line with published data (21, 25), anti–TGF-β and several Abs binding to L-TGF-β/GARP inhibited Smad3 phosphorylation in stimulated Treg, which indicated that these Abs prevented the release of active TGF-β from cell surface–bound L-TGF-β/GARP, and interfered with the conformational change necessary for activation or, in case of anti-TGF-β, directly neutralized active TGF-β. However, these Abs did not influence the suppressive activity of Treg in vitro. Other groups have also failed to identify a major contribution of TGF-β to the suppressive capacity of Treg (41). In contrast, Cuende et al. (21) demonstrated that anti–TGF-β as well as the anti–L-TGF-β/GARP Abs MHG-8 and LHG-10 reduced the immunosuppressive activity of a Treg clone in a suppression assay in vitro.

We were also not able to confirm a prominent role of GARP in other functions of Treg, such as FOXP3 induction in Tcon or stability of Treg. It has been shown previously that activated Treg can induce FOXP3 expression in Tcon during coculture (10, 36). In the study with human cells, activation of Tcon in the absence of Treg did not lead to strong induction of FOXP3. In contrast, we and others have observed that FOXP3 expression was enhanced in human Tcon after in vitro stimulation and specifically in Tcon that have divided (Fig. 5A and Ref. 42). In our experiments the increase was most likely not caused by residual TGF-β in the medium, as we could not detect TGF-β by ELISA in the medium or serum used. Therefore, interpretation of FOXP3 expression in human Tcon is complex, as it can be induced by TGF-β as well as by stimulation/proliferation. In our study, coculture of Tcon with Treg inhibited proliferation of Tcon (Fig. 5A, 5B). In line with the lower fraction of cells that had divided, the percentage of FOXP3^+^ Tcon was reduced. This reduction of FOXP3 expression might have overlaid a potential induction by Treg-derived TGF-β. Indeed, the slight reduction of FOXP3 expression in the presence of anti–TGF-β might support this speculation (Fig. 5B). As we could not detect a strong TGF-β–dependent effect in the Tcon/Treg coculture, an effect of the anti–L-TGF-β/GARP Abs was not expected and was not observed (Fig. 5B). A different potential mechanism for Tcon to Treg conversion has been described in which FOXP3 expression was induced in Tcon by activation of TGF-β on their cell surface by interaction of L-TGF-β/GARP with α_v_β_8_ (13). L-TGF-β/GARP was expressed on Tcon, and the model suggested that activated TGF-β signaled to Tcon. This mechanism has been mainly studied in mouse T cells. We and others have not found expression of GARP protein on human Tcon (Fig 2 and Ref. 10), so we did not further investigate this mechanism.

While we detected only low levels of TGF-β (<80 pg/ml) in the supernatant of stimulated Treg (Fig. 2E), we demonstrated strong immunosuppressive effects of recombinant TGF-β on T cells only at much higher levels (>1 ng/ml). This finding may suggest a role for cell surface–bound TGF-β. Our experiments did not directly differentiate between both mechanisms. However, compared with recombinant TGF-β (1 ng/ml), stimulated Treg induced only a very low signal in a TGF-β reporter cell line (data not shown), questioning the presence of high levels of cell surface–bound active TGF-β. The published structural model of the α_v_β_8_/L-TGF-β/GARP/TGF-βR2 signaling complex (13) predicted geometric constraints leading to potential inaccessibility of target epitopes for anti–TGF-β or anti-GARP Abs. Accordingly, an anti-β_2_ Ab was more potent in blocking signaling by α_v_β_8_-activated TGF-β than Abs to TGF-β or GARP. This finding might be a good explanation for the lack of effect of anti–TGF-β Abs in some Treg assays. In our experiment, TGF-β signaling was also dependent on integrins, but the Abs to TGF-β or L-TGF-β/GARP were able to block signaling (Fig. 3A). We have not performed a detailed comparison of Ab potencies. It will be interesting to investigate in more detail in which physiological situations TGF-β signaling is induced by either released TGF-β, or by cell-bound active TGF-β from the same cell or from a neighboring cell.

These conflicting results fit into the long-standing debate about the role of TGF-β for Treg suppression (14), and, accordingly, the relevance of GARP for Treg is still not fully understood (18, 19). The outcome seems to be highly dependent on the context and experimental conditions, and differences between mouse and human biology have been reported (41). Physiological or pathological conditions may differ considerably from the experimental systems, thus making translation from cell culture to patients challenging. For example, Treg/Tcon ratios are presumably much lower in vivo than those that are usually used in vitro, and additional cell types, such as dendritic cells may be involved.

Treg-specific GARP knockout or treatment with an Ab against mouse TGF-β1/GARP complexes did not inhibit tumor growth in mouse models (19, 23). However, combination of anti-TGF-β1/GARP with anti–PD-1 treatment induced regressions of tumors that were resistant to anti–PD-1 alone (23), demonstrating a therapeutic potential of such Abs and indicating that blockade of more than one suppressive pathway may be necessary. In a similar approach, recently bintrafusp alfa, a bifunctional fusion protein designed to simultaneously block TGF-β and PD-L1, has been tested in the clinic. The results were disappointing, and the respective phase III trial has been discontinued (43).

GARP is also expressed on platelets, which are an additional source of TGF-β in vivo. For tumor therapy it may be equally important to inhibit platelet-associated as well as Treg-associated TGF-β/GARP (23, 24). Platelets may also serve as a sink for a therapeutic Ab via target-mediated disposition.

Taken together, the GARP/TGF-β axis represents an exciting biological pathway that can be targeted pharmacologically in different ways. However, further studies are necessary to understand its complexity and to unleash its therapeutic potential.

## Supplementary Material

Supplemental Figures 1 (PDF)
