# Supplementary material for: Anti-GARP Antibodies Inhibit Release of TGF-β by Regulatory T Cells via Different Modes of Action, but Do Not Influence Their Function In Vitro
Source: Immunohorizons. 2023 Mar 16;7(3):200–12. doi: 10.4049/immunohorizons.2200072 (PMC10563435; doi:10.4049/immunohorizons.2200072)
Supplement: Supplemental Figures 1 (PDF) [file IH_2200072_Supplemental_1.pdf]

## Supplementary Figure 1

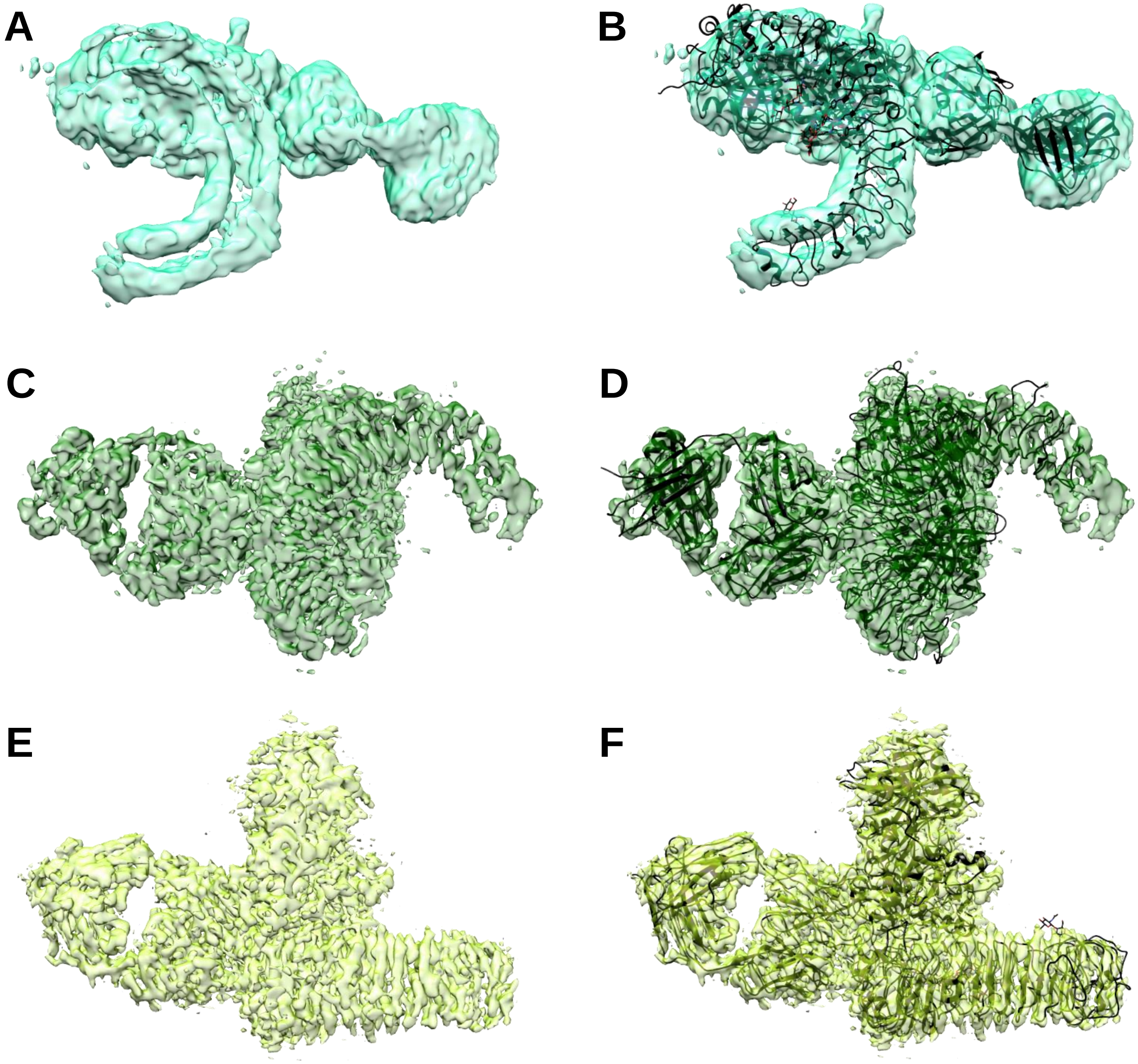


**Cryo-EM maps and fitted models.** Cryo-EM maps (**A, C, E**) and models (cartoon representation) fitted in the corresponding maps (**B, D, F**) of the GARP/L-TGF-β in complex with **A, B** Fab 28G11 (cyan) at 7.5 Å resolution, **C, D** LHG-10 (lemon green) at 2.7 Å resolution, and **E, F** 12G2B4 (dark green) at 3.4 Å resolution. All Fabs offer good model to map correlation considering the corresponding resolution.

# Supplementary Figure 2

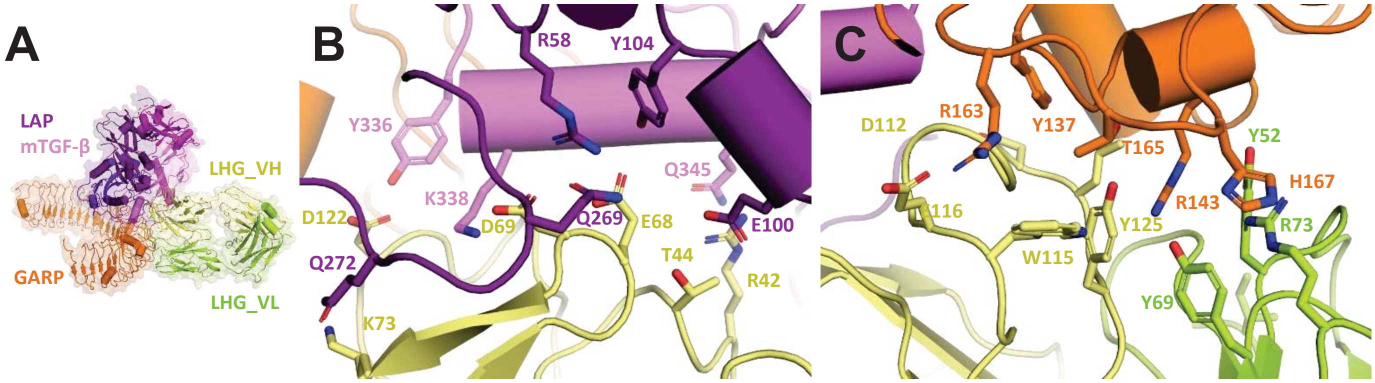


**Detailed representation of the GARP/L-TGF-β-LHG-10 epitope and paratope.** **A** Schematic overview scheme of the GARP/L-TGF-β-LHG-10 complex. **B** The binding epitope of LHG-10 (yellow and lemon green) on LAP (dark purple) consists of residues 269-273, 58, 100, 104 and residues 336-338, 345 on mTGF-β (light purple). **C** GARP (orange) residues 137, 140-143, 161-163, 165 and 167 interact with the light (lemon green) and heavy (yellow) chain of the variable regions of Fab LHG-10.

## Supplementary Figure 3

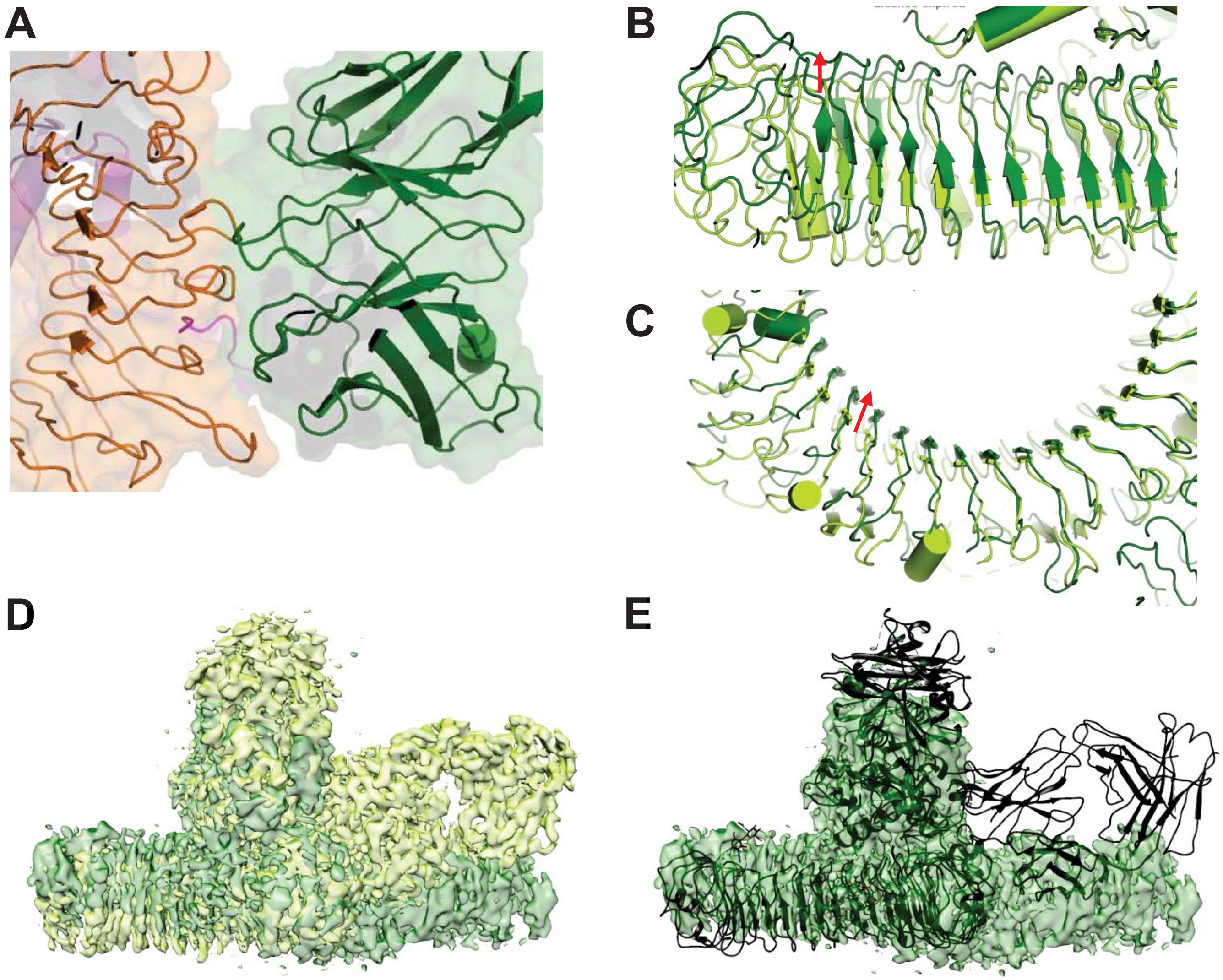


**Conformational rearrangements upon binding of Fab 12G2B4. A** 12G2B4 (dark green) binds exactly at the point where the horseshoe fold of GARP (orange) is completed by a β-strand addition of LAP (magenta). Here, the β -sheet at the N-terminus of LAP intercalates into the β -sandwich of GARP. **B** Upon binding of 12G2B4 a conformational rearrangement of GARP is induced. In comparison to the LHG-10 bound structure (lemon green), the C-terminal portion of 12G2B4 bound GARP (dark green) moves up towards L-TGF-β and **C** In addition, the curvature of the horseshoe folds increases in this region, resulting in a slight dislocation compared to the LHG structure. **D** These conformal changes are also clearly visible in the EM density of the structure, as shown by the superposition of the maps of LHG-10 (lemon green) and 12G2B4 (dark green)**. D** Overlay of 12G2B4 cryo-EM map and LHG-10 model (black), aligned on GARP N-terminal portion and L-TGF-β.

**Supplemental Table 1: Data collection, processing and refinement statistics for GARP-L-TGF-β cryo -EM structures in complex with different Fabs.**

| | | | |
|---|---|---|---|
| **Experimental data** | | | |
| Protein | GARP - L-TGF-β | GARP - L-TGF-β | GARP - L-TGF-β |
| Fab | LHG-10 | 28G11 | 12G2B4 |
| PDB /EMBD ID | EMD-16460<br>8C7H | EMD-16456 | EMD-16459 |
| **Data Collection and Processing** | | | |
| Microscope | FEI Titan Krios | FEI Titan Krios | FEI Titan Krios |
| Voltage (kV) | 300 | 300 | 300 |
| Camera | Gatan K2 Summit | Gatan K3 | Gatan K3 |
| Exposure time (s) | 8 | 8 | 8 |
| Total Dose ($e^-/Å^2$) | 40 | 40 | 40 |
| Defocus range (µm) | 0.8-2.8 | 0.8-2.8 | 0.8-2.8 |
| Pixel size (Å) (calibrated) | 1.08 | 0.83 | 0.83 |
| Symmetry imposed | C1 | C1 | C1 |
| Number of micrographs | 2842 | 3354 | 8289 |
| Initial particle number | 2,837,425 | 3,639,216 | 2,602,167 |
| Final particle number | 418,886 | 488,857 | 613,675 |
| Map resolution (Å) | 2.7 | 4.3 | 3.4 |
| FSC threshold | 0.143 | 0.143 | 0.143 |
| **Refinement** | | | |
| Model composition | | | |
| Protein (residues) | 9172 | | |
| Ligands | BMA:3; NAG:6 | | |
| RMSD bond length (Å) | 0.003 | | |
| RMSD bond angles (°) | 0.661 | | |
| Rotamer outliers (%) | 0.42 | | |
| ADP (B-factor) (min/max/mean) | | | |
| Protein | 3.37/159.21/68.62 | | |
| Ligand | 16.10/94.14/53.72 | | |
| Ramachandran plot | | | |
| Favoured (%) | 95.61 | | |
| Allowed (%) | 4.39 | | |
| Outliers (%) | 0.00 | | |
| All-atom clashscore | 8.20 | | |
| MolProbity score | 1.75 | | |
